# Virulence factors in multidrug (MDR) and Pan-drug resistant (XDR) *Pseudomonas aeruginosa*: a cross-sectional study of isolates recovered from ocular infections in a high-incidence setting in southern India

**DOI:** 10.1186/s12348-021-00268-w

**Published:** 2021-09-28

**Authors:** Poonam Naik, Suchita Pandey, Satyashree Gagan, Sudeshna Biswas, Joveeta Joseph

**Affiliations:** 1 Jhaveri Microbiology Centre, Brien Holden Eye Research Centre, L. V. Prasad Eye Institute, Hyderabad, India; 2 Research Scholar, Manipal Academy of Higher Education, Manipal, India

**Keywords:** Endophthalmitis, Multi-drug resistance, *Pseudomonas aeruginosa*, Virulence factors

## Abstract

**Background:**

Global concerns have been raised due to upward trend of Multi-drug Resistant (MDR) *Pseudomonas aeruginosa* reports in ocular infections. Our aim was to characterize the virulence determinants of MDR *P. aeruginosa* causing ocular infections.

**Methods:**

*P. aeruginosa* strains were isolated from 46 patients with conjunctivitis (2), endophthalmitis (11) and active keratitis (25) seen at our Institute, between 2016 and 2020. The isolates were identified by Vitek-2 and characterized based on growth kinetics, biofilm formation, motility, pyoverdine and pyocyanin production, phospholipase and catalase activity, urease production along with expression of exotoxins (exo-A, exo-U and exo-S) and correlated to its antibiotic profiles.

**Results:**

Of the 46 *P. aeruginosa* isolates, 23 were MDR and were significantly (*p* = 0.03) associated with older (> 65) patients, along with higher production of pyoverdine (58.3%), pyocyanin (30.4%), phospholipase (91.6%) and protease (62.5%) activity, formed strong biofilms and exo-A (30.4%). No significant relation between motility, urease and catalase production with antibiotic susceptibility was observed. Heatmap and PCoA analysis confirmed this unique virulence profile associated with *MDR-PA* strains.

**Conclusion:**

Phenotypic characteristics of *P.aeruginosa* might be responsible for increased colonization and antibiotic resistance observed in vivo and understanding these differences may lead to development of clinical guidelines for the management of MDR infections.

## Introduction

*Pseudomonas aeruginosa* is the most common Gram-negative isolate causing vision-threatening ocular infections including conjunctivitis, keratitis, orbital cellulitis and endophthalmitis [1–4]. *P. aeruginosa* has low nutritional requirements and can high tolerance to a range of physical conditions, thus making it more pathogenic. *P. aeruginosa* infections are very difficult to eradicate due to their intrinsic resistance to antibiotics [5], in addition, to various virulence factors like flagellin and lipopolysaccharide, as well as secreted products such as cytotoxins [6, 7], elastase [8, 9], alkaline protease [10, 11], protease IV [12], as well as its invasiveness and increased colonization has been reported to contribute to its pathogenicity [13–16]. The presence of these secretion toxin-encoding genes in clinical isolates from different infections is associated with differences in bacterial virulence [17, 18] and clinical outcomes [19]. Proteases is said to contribute to pathogenesis through destruction of connective tissue and degradation of host immunological factors [20] in patients with keratitis. Previous reports have also suggested that pyocyanin and pyoverdine not only contribute to the increased colonization in the lungs of patients with cystic fibrosis, it also interferes with cell respiration, calcium homeostasis and prostacyclin release from lung endothelial cells as well [21]. Another important factor is the ability of *P. aeruginosa* in biofilm formation beginning with the involvement of redundant planktonic cells in a complex and highly regulated physicochemical and biological signalling and thereby confers resistance and protects the bacteria against host immune responses [22–27]. Increasingly, clinical isolates of *Pseudomonas* are exhibiting multiple resistance to antibiotics and becoming pan drug resistant (XDR) [28]. The Emergence of resistant (MDR and XDR) strains in ocular settings, particularly in tropical countries like ours [29], has become a major problem, leaving few alternatives for treatment of these patients [30]. This increase in incidence of MDR and XDR infections is also associated with increased morbidity, mortality, and costs [31, 32]. The aim of our present study was to screen *P. aeruginosa* strains isolated from ocular infections, for their potential to produce various phenotypic virulence factors and correlating them with biofilm formation and their antibiotic susceptibility profile. The hypothesis tested was that MDR and XDR *P. aeruginosa* strains possess distinguishing virulence characteristics in comparison with *P. aeruginosa* that were susceptible to most antibiotics.

## Materials and methods

The study was approved by the Institutional Review Board of the L V Prasad Eye Institute, and was performed in accordance with the ethical standards as laid down in the Declaration of Helsinki. Clinical and microbiology records were retrospectively reviewed for patients who were evaluated at our institute and diagnosed with culture-confirmed infections [keratitis, endophthalmitis, cellulitis, conjunctivitis] due to *P. aeruginosa*.

### Bacterial isolates

*Forty-six, Pseudomonas aeruginosa* isolates from various clinical samples from patients with eye infections during the period of April 2016 to March 2020 were included in the study. The preserved isolates were revived and their identification was confirmed by standard microbiological and biochemical methods based on gram staining technique and colonial morphology in addition to Vitek 2 compact system testing using GN strips (bioMérieux).

### Antibacterial susceptibility testing

For antibiotic susceptibility testing, minimum inhibitory concentration (MIC) was determined using E-test strips (Himedia) or VITEK® 2 AST cards according to the manufacturer’s protocol [33] and this included ciprofloxacin, moxifloxacin, gatifloxacin, ofloaxacin, ceftazidime, gentamicin, tetracycline, amikacin, tobramycin, piperacillin, norfloxacin, colistin and imipenem. All results were compared to the Clinical and Laboratory Standards Institute (CLSI) interpretative guidelines and the isolates were classified as susceptible (S), susceptible dose dependent (SDD), and resistant (R) [34]. However, for the purpose of analysis, SDD isolates were clubbed with susceptible isolates (S-PA). Multiple drug resistant (MDR) phenotype was assigned for strains that was resistant to ≥3 classes of antibiotics. The *P. aeruginosa* ATCC 27853 strain (American Type Culture Collection), was used as the quality control. The *P. aeruginosa* phenotype was defined as MDR and XDR according to the international expert proposal for interim standards guidelines [18].

### Growth curve analysis

The in vitro growth rate was assessed by diluting 1 × 10^6^ CFU/ml of each isolate in BHI broth, on a rotary shaker at 160 rpm for 48 h. At periodic intervals, serial dilution of each culture was plated on an antibiotic-free Mueller Hinton agar and the number of CFUs [colony-forming units] was counted after 24 h incubation at 37 °C.

### Phenotypic characterization of *P. aeruginosa* isolates

All phenotypic assays (described below) were performed as three independent biological replicates unless otherwise specified. The *P. aeruginosa* ATCC 27853 strain was used as the positive control to assay motility, biofilm formation and secreted virulence factors.

### Measurement of Pyoverdine production and qualitative assessment of pyocyanin

Pyoverdine production by *P. aeruginosa* was assayed spectrophotometrically as previously described [35]. Briefly, the isolates were cultured in MHB to late stationary phase (OD of ‘3 was recorded at 600 nm). The cultures were then centrifuged at 10,000×g for 2 min and the supernatant were normalized for differences in cell density, and the absorbances measured at 405 nm. The concentration of pyoverdine was then calculated using the extinction coefficient as follows: Molar concentration = Absorbance/Extinction coefficient (1.9 X 10^− 4^ M^− 1^ cm^− 1^).

Pyocyanin production was assessed using a qualitative method as described earlier by Alonso et al. [36]. Inoculated plates were incubated at 37 °C for 24 h. Colonies appearing Dark green or blue in colour were considered to be pyocyanin producers.

### In vitro microplate *biofilm assay*

The ability of *P. aeruginosa* strains to develop biofilm was determined on 96-well microtiter plates with crystal violet (CV) staining method as previously described [37]. Briefly, each *P. aeruginosa* strain was cultured on Mueller Hinton agar overnight, and a colony of each isolate was suspended in brain heart infusion (BHI) broth and incubated at 37 °C for 4 h. Bacterial suspensions were then adjusted to an optical density of 0.1 at 600 nm and added to flat-bottomed 96-well sterile culture plates. Following a 48 h incubation period at 37 °C, non-adherent bacteria were removed by washing and stained with 0.1% crystal violet. Following incubation at 30 min, the plates were again washed and ethyl alcohol was added each well, after which the absorbance was measured at 590 nm. The OD590 values were then normalized with initial OD600 values to account for differences in bacterial growth and biofilm was classified as weak, moderate or strong as described earlier [38].

### Motility assay

Motility of the strains was determined in motility medium, which consists 1 (w/v) tryptone, 0.3% yeast extract, 0.5% NaCl and 0.3% agar. Plates were stab-inoculated from overnight cultures and incubated at 37 ^°^C for 24 h. Each strain was assayed in triplicate. The diameter of the circular zone of growth was measured and expressed as a mean value in mm. An isolate showing a change of ≥ 10% was considered highly motile while the rest were categorized as weakly motile or non-motile.

### Catalase activity

Determination of the enzyme’s presence in the bacterial strains was done using a sterile loop to place a small amount of growth onto the base of a Petri dish, followed by a drop of hydrogen peroxide and covering the Petri dish with a lid [39]. The development of effervescence indicates a positive result.

### Extracellular protease activity

Qualitative protease activity was evaluated by streaking bacteria onto modified basal medium supplemented with 6.2 g/L skim milk protein and incubated for 48 h at 37 °C. Proteolytic activity was demonstrated by a clearing zone (> 12 mm) surrounding the bacterial growth.

### Phospholipase [plc] activity assay

Haemolytic activity of the isolates was analysed by presence of clear halos around growth of the organism on blood agar plates after 24 h incubation at 37 °C [40].

### Urease test

Urease activity was determined by inoculating the strains on to Christensen’s urea agar slope (Oxoid, Thebarton, South Australia) and incubated at 21 °C with protection from light for seven days [41]. Following incubation, the slopes were examined visually for a colour change to pink which was considered to be a positive result, and no change (or a yellow colour) was considered a negative result.

### DNA extraction and genotypic detection of virulence genes

Strain DNA was extracted using the QIAamp DNA Mini Kit (50) (QIAGEN) following the manufacturer’s instructions. DNA was eluted in 30 μl of elution buffer. The genes, exotoxin S (exoS), exotoxin U (exoU), exotoxinA (exoA) were amplified using the specific primers as described earlier [41, 42]. The PCR protocol involved initial denaturation step at 95^°^C for 10 min, followed by 40 cycles of 94^°^C for 2 min, annealing (30s at 57 to 65 °C) and 72^°^C for 1 min and the final extension step at 72^°^C for 5 min.

### Statistical analysis

The data were processed on spreadsheets and all statistical analyses were performed using GraphPad prism [5.0]. Phenotypes were treated as either categorical or continuous variables and analysed as appropriate. Wherever applicable all comparisons were evaluated using either χ^2^ test or unpaired student t test. The CFU were represented as mean CFU ± SE of 23 strains in each group at indicated time points and a *P* value of less than 0.05 was considered significant.

## Results

Forty-six isolates of *P. aeruginosa* were obtained from various clinical specimens [corneal, conjunctival and scleral scraping, corneal buttons and vitreous fluids] during the study period and the clinical and demographic details are elaborated in Table 1. Clinical diagnosis included conjunctivitis [3], scleritis [1], endophthalmitis [12], cellulitis [2] and active keratitis [28] diagnosed at our institute, during the study period. Of these isolates, 23 [50%] were found to be MDR which also included 9 (19.5%) XDR strains. As expected, *MDR-PA* was significantly associated with poor visual outcome and prognosis (*p = 0.03*). Interestingly, these MDR strains were significantly (*p* = 0.03) associated with older (> 65) patients. Out the 23 patients infected with *S-PA* strains, 2 patients underwent evisceration who were diagnosed with post enucleation socket syndrome and microbial keratitis. Additionally, 2 patients with *MDR-PA* caused microbial keratitis and 2 patients with *MDR-PA* induced endophthalmitis cases lead to evisceration.

**Table 1:** Demographic and microbiological data of the patients included in the study

Categories	Susceptible(*S-PA*) *N* = 23(%)	Multi-drug resistant (*MDR-PA*) *N* = 23(%)	*p*-value
Gender			
Male	12 (52.17%)	13 (56.52%)	^a^1
Female	11 (47.82%)	10 (44.47%)	
Mean Age ± SD (years)	39.29 ± 20.05	49.34 ± 18.67	^a^0.11
(Range)	(1–80)	(4–73)	
Disease			
Microbial keratitis	14 (60.86)	11 (47.83)	
Endophthalmitis	3 (13.04)	8 (34.78)	
others	6 (26.08)	4 (17.39)	
VA			
> (20/200)	12	16	**0.03**
> (20/20) - (20/200) <	6	1	
Evisceration	2	4	
Unknown	3	2	
Polymorphs			
(0–1,0-3,0–5)	8	5	0.2
(0–10, plenty)	13	17	
Unknown	2	1	

a: Pearson’s chi-squared test

### Growth kinetics differences between MDR-PA and S-PA

**Fig. 1:**
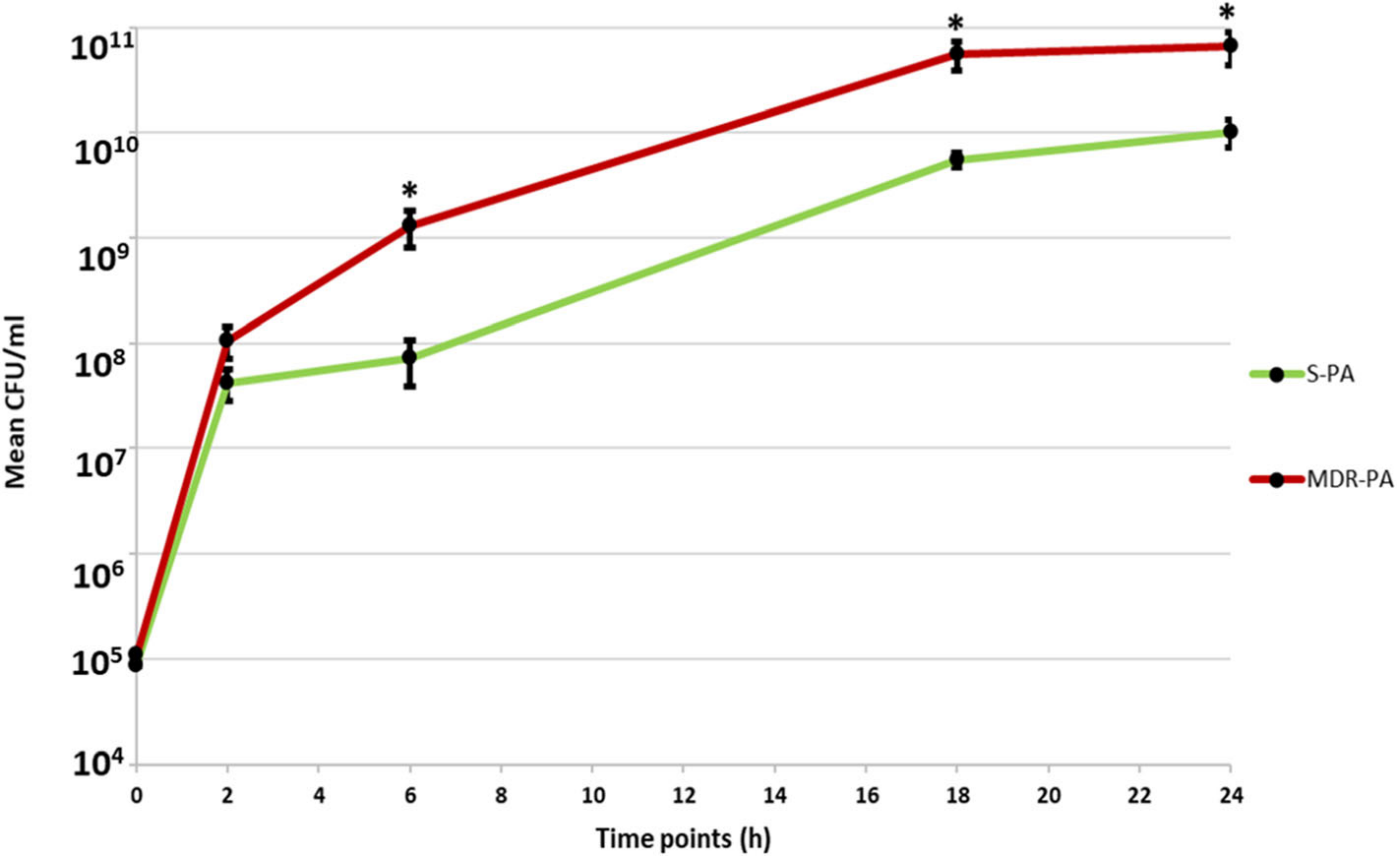
Clinical isolates of *P. aeruginosa* were plated on MHA at indicated time points. Bacterial load and viability were enumerated by plate count method. Student’s test was used for the statistical analysis and data are represented as the mean colony forming units (CFU ± SD) from three sets of independent experiments. *p < 0.05

To determine potential differences in growth kinetics, the colony forming units on MHA plates were determined for each strain, by plotting the mean CFU of all *S-PA* and *MDR-PA* strains at different time points (0,2,6,18,24 h). At 6 h, 18 h and 24 h significant differences in colony counts were observed between the drug susceptible strains vs multi-drug resistant strains as shown in Fig. 1. The mean CFU at 6 h of *S-PA* Vs *MDR-PA* strains were (7.82 × 10^7^ Vs 1.31 × 10^9^*, p = 0.01*), at 18 h (5.22 × 10^9^ Vs 5.6 × 10^10^*, p = 0.01*) and 24 h (1.01 × 10^10^ Vs 6.65 × 10^10^*, p = 0.03*). The plotted growth curve suggests that *MDR-PA* strains demonstrate significantly higher growth kinetics at later time points compared to *S-PA* strains.

### Phenotypic characterization of S-PA and MDR-PA strains

**Fig. 2:**
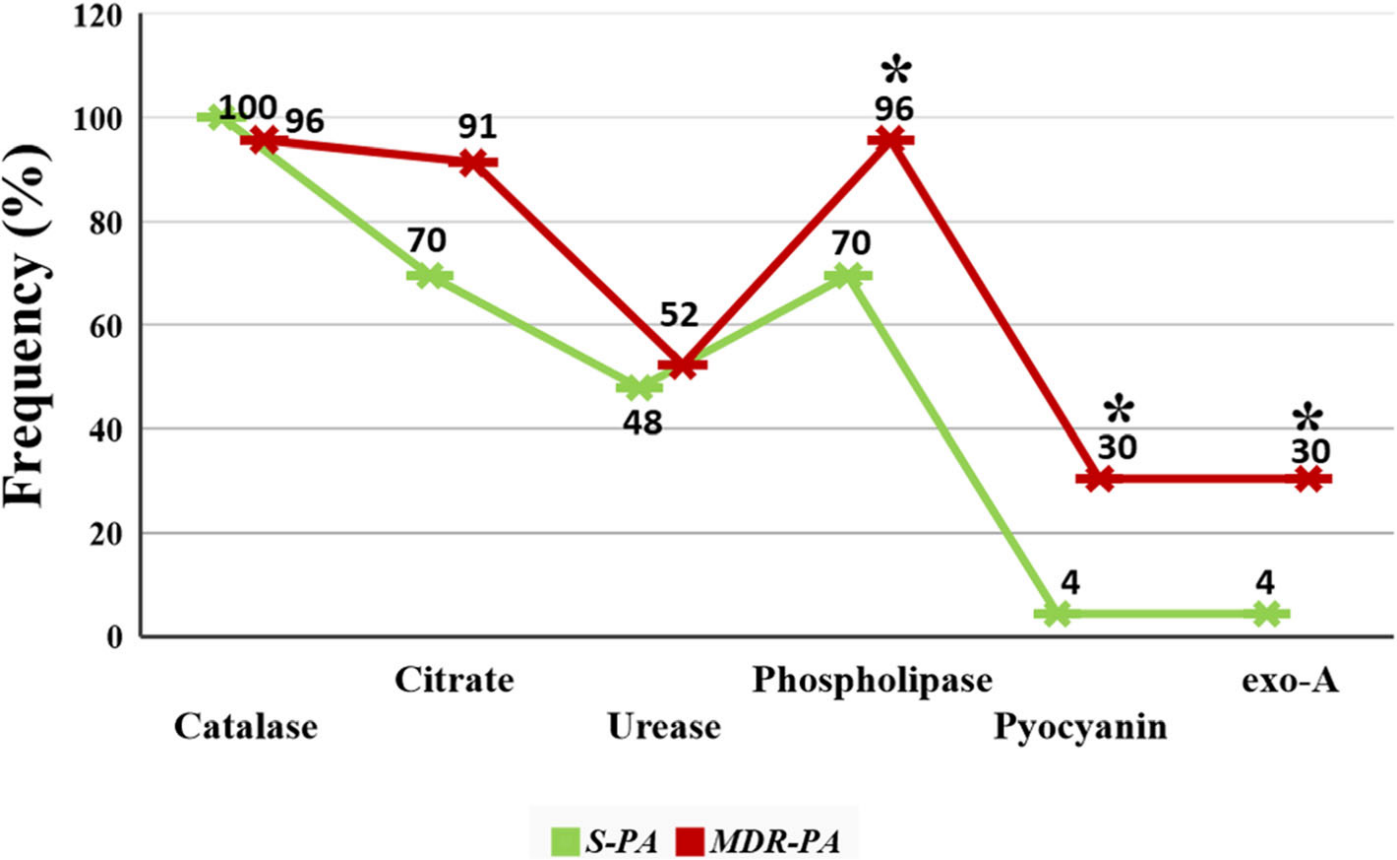
Graphic representation of frequency of enzymatic virulence factors (catalase, citrate, urease,phospholipase, pyocyanin and exo-A) expression. Pearson’s Chi-square test was used for the statistical analysis and data are represented as the mean from three sets of independent experiments. **p ≤ 0.01, *p < 0.05

Almost all the strains in both *S-PA* and *MDR-PA* group were catalase positive. While 11/23 (48%) *S-PA* strains were urease positive and 12/23 (52%) of the *MDR-PA* group were positive for urease. Similarly, for citrate production, 91% of *MDR-PA* and 70% of the *S-PA* isolates were positive. Additionally, phospholipase C activity was significantly (chi square test, *p = 0.02*) higher in *MDR-PA* (96%) strains when compared with the *S-PA* strains (70%) as shown in Fig. 2, suggesting a potential role in strain virulence. We found that 7 (30.4%) out of the 23 *MDR-PA* isolates produced pyocyanin while only one *S-PA* (4.3%) isolate produced pyocyanin pigment and there is significant association of pyocyanin production with multidrug resistant (*p = 0.02*).

**Fig. 3:**
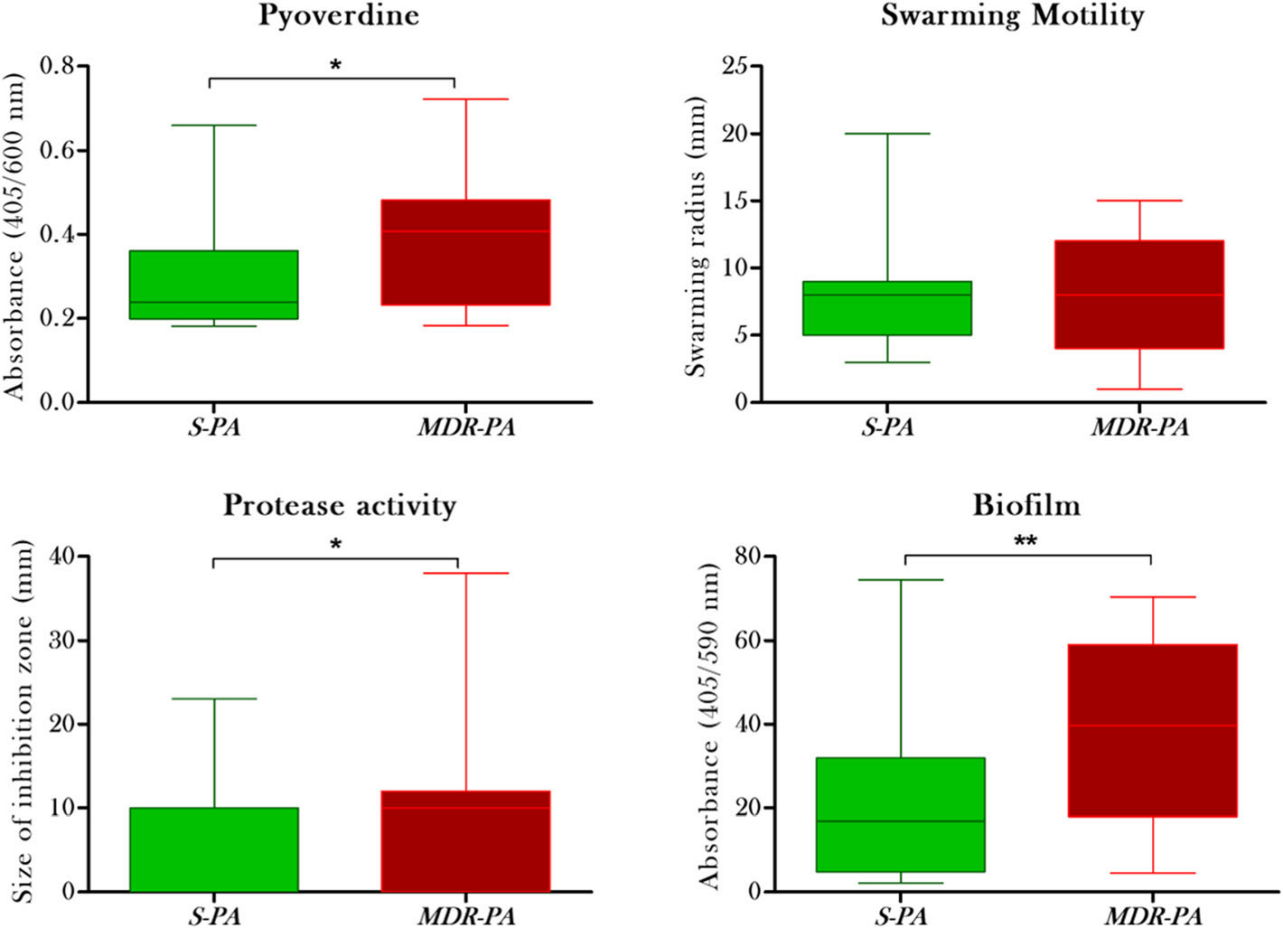
(**A**) Quantitative analyses of the pyoverdine production by spectrophotometer analysis are shown in the box-plot. (**B**) Swarming activity were quantitively analysed by measuring the distance moved by the isolates (**C**) Quantitative analysis of protease production by *S-PA* and *MDR-PA* strains (**D**) Quantitative analyses of the results from crystal violet retention assays are shown in the box-plot. Unpaired Student’s test was used for the statistical analysis and data are represented as the mean ± SD from three sets of independent experiments. **p ≤ 0.01, *p < 0.05

**Fig. 4:**
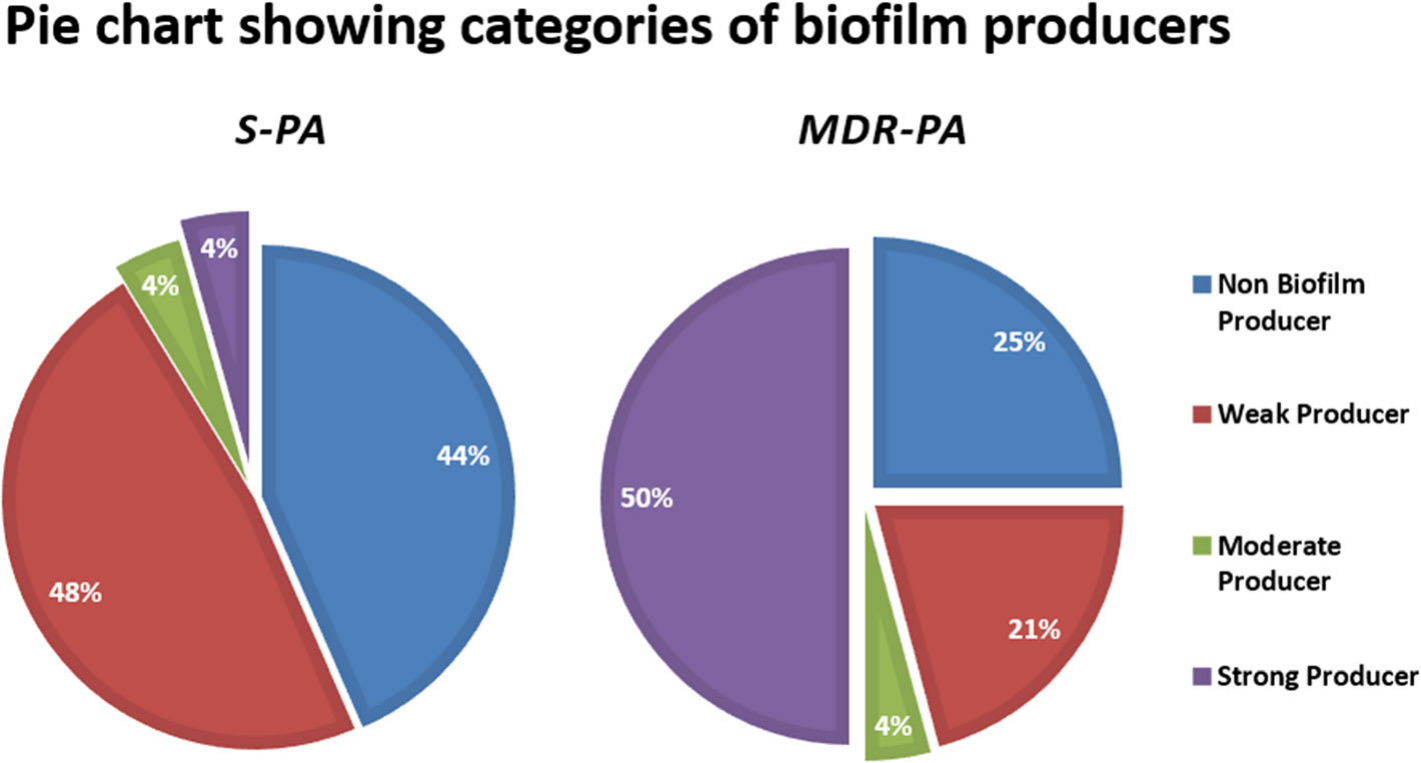
Graphic representation of a biofilm production by *S-PA* and *MDR-PA* group. 50% of the *MDR-PA* isolates were strong producer whereas in case of *S-PA* only 4% isolates were strong biofilm producer

Looking at the pyoverdine production, the mean absorbance (Fig. 3A) was again significantly higher in *MDR-PA* strains, compared to the *S-PA* (0.39 ± 0.03 nm Vs 0.28 ± 0.02 nm, *p = 0.01*) indicating its role in virulence. Though the *MDR-PA* strains showed increased motility compared to the *S-PA* group, this difference (7.82 ± 0.90 mm vs 8.04 ± 0.04 mm, *p* = 0.8) was not statistically significant (Fig. 3B), however, 50% strains belonged to the XDR group. Similarly, protease activity was significantly higher in *MDR-PA* group when compared to *S-PA* group (9.48 ± 0.96 mm vs 5.17 ± 1.88 mm, *p = 0.02*) (Fig. 3C) of which, 80% strains belonged to the XDR phenotype. Analysis of biofilm ability revealed an increased production of biofilm by *MDR-PA* group (39.04 ± 4.88 nm vs 21.10 ± 4.19 nm, *p = 0.007*) as shown in Fig. 3D. Additionally, among the *S-PA* strains, 48% formed weak biofilms, 4% were moderate, 4% were strong biofilm formers (Fig. 4) and 44% were non biofilm producers. In comparison, in the *MDR-PA* group, 50% of the strains were strong biofilm producer, while 25% were weak biofilm producers. Next, we compared *XDR-PA* and *MDR-PA* group to find out if there is any association of XDR with the virulence traits. In our present study we didn’t find any significant association of *XDR* with virulence factors (biofilm; *p = 0.51*, urease; *p = 0.13*, protease; *p = 0.42*, motility; *p = 0.40*, pyoverdine; *p = 0.71*, citrate; *p = 0.84*) compared to the *MDR-PA* isolates.

This trend suggests a significant association of biofilm production with multi-drug resistance. Summarizing the virulence factors, we observe that production of catalase, urease and swarming ability does not have any correlation with the level of antibiotic resistance (Table 2) whereas pyoverdine production, biofilm ability, protease and phospholipase activity were significantly associated with antibiotic resistance and virulence.

**Table 2:** Frequency and Statistical correlation of different virulence factors among Resistant and susceptible *P. aeruginosa* strains

Phenotypic character	***S-PA***	***MDR-PA***	***P***-value	OR (95% CI)
**Pyoverdine**	**4**	**14**	^**a**^0.01*	6.65 (1.7–25.6)
**Pyocyanin**	**1**	**7**	^**a**^0.02*	0.1 (0.01–0.93)
**Biofilm**	**13**	**18**	^**a**^0.007*	2.23 (0.6–7.9)
Swarming	4	8	^**a**^0.12	–
Catalase	23	22	–	
**Protease**	**5**	**15**	^**a**^0.02*	3.8 (1.1–12.8)
**Phospholipase**	**16**	**22**	^**a**^0.01*	5.8 (1.0–31.5)
Urease	11	12	^**a**^0.88	–

a:Pearson’s chi-squared test, *P* probability, *OR* Odds Ratio, *CI* confidence interval

Further, we compared association of anatomical site with the virulence factor. Out of the 25 samples 14(56%) were antibiotic susceptible and 11(44%) were multi-drug resistant. We did not observe any difference in the virulence factor (biofilm; *p = 0.20*, urease; *p = 0.46*, phospholipase; *p = 0.12,* protease; *p = 0.32*, motility; *p = 0.57*, pyoverdine; *p = 0.30, citrate; p = 0.4*) between the *S-PA* isolates and *MDR-PA* isolates.

**Fig. 5:**
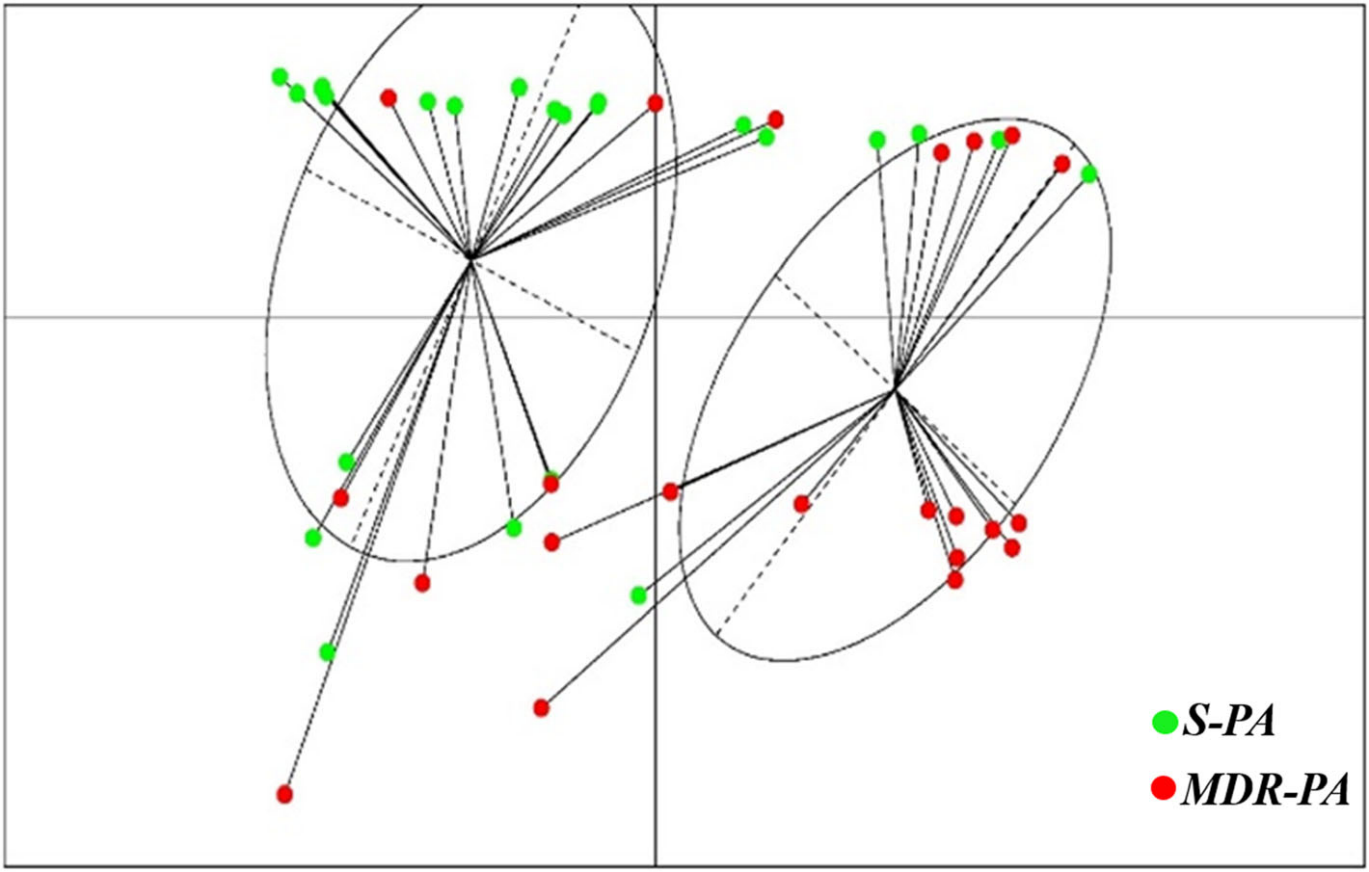
Principal coordinate analysis (PCoA) plot of *S-PA* (left) and *MDR-PA* (right) and the high-resolution display of red square indicates *MDR-PA* isolates while the green dot represents *S-PA* isolates. PCoA plot was generated from the values obtained from Pyoverdine, biofilm, protease and motility test (*n* = 23 in each group)

**Fig. 6:**
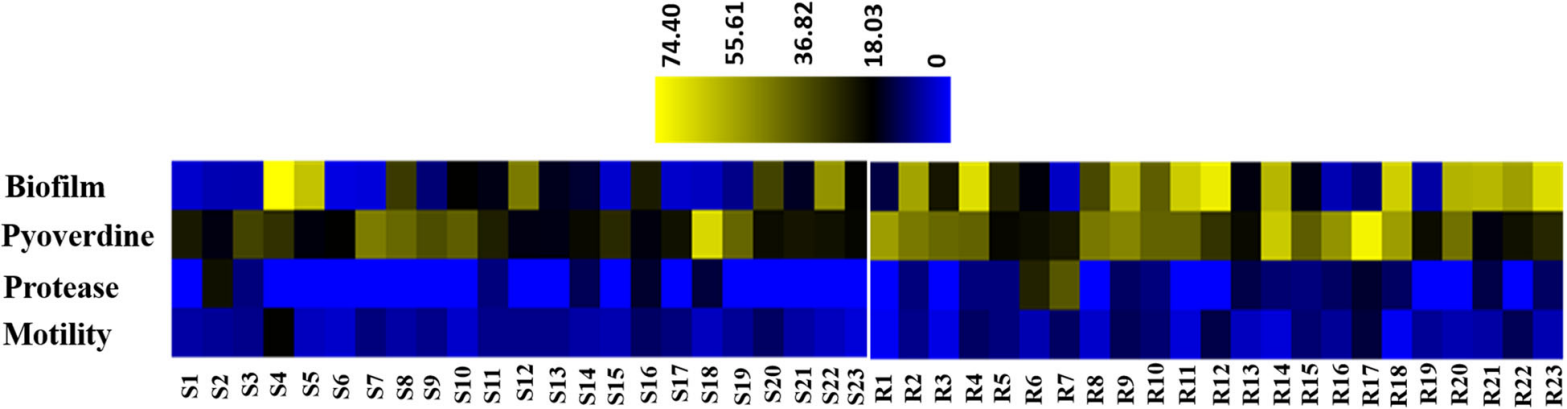
Heat map representing virulence factors of ocular clinical isolates of *P. aeruginosa*. The Virulence factors for 46 clinical isolates were tested by different methods specified earlier. Heat map was constructed to compare the *S-PA* and *MDR-PA* isolates. S1-S23 denotes drug susceptible strains, R1-R23 denotes *MDR-PA* strains. Blue represents a lower level of expression and yellow represents a higher level of expression

To further confirm our results, we carried out a principal Coordinate Analysis to explore and to visualize dissimilarities of these virulence factors amongst the *S-PA* and *MDR-PA* group. Application of this method to our data showed that the samples could be divided into two principal groups: one consisting primarily of *S-PA* strains (Fig. 5, cluster 1) and second independent clusters of mainly *MDR-PA* strains (Fig. 5, clusters 2). Red dot indicates *MDR-PA* and green indicates *S-PA*. The plot shows the Euclidian distance between the two groups based on how the two groups can be distinguished. We further constructed a heat map to depict the relative virulence factors expressed by each strain as shown in Fig. 6. The relative colour intensity of heat map gave a good overview of the profile differences between the two groups and demonstrates that the virulence factors are more strongly associated with the *MDR-PA* clinical isolates.

### Detection of exotoxins in S-PA and MDR-PA strains

Our study found that the exoA was present in 1/23(4.3%) and exoS 2/23(8.69%) of the S-PA isolates while we couldn’t detect exoU gene in any of the *S-PA* isolates. In case of *MDR-PA* isolates, exoA was present in 7/23 (30.4%) of the isolates, being significantly associated with multidrug resistance (*p = 0.02*). However, exo-S was not detected in any of the *MDR-PA* strains and only 2 strains showed the presence of exo-U (8.69%).

## Discussion

*P. aeruginosa* is a predominant Gram-negative bacterium causing ocular infections [43]. In the present study, *P. aeruginosa* strains were obtained from corneal ulcers, vitreous fluids and purulent discharge from patients diagnosed at our institute with various infectious conditions. Characterization of the armor of *P. aeruginosa* virulence factors is essential to understand the pathogenesis of this opportunistic pathogen as well as helps in exploring new antimicrobial strategies in MDR strains. *P. aeruginosa* is also known to secrete a number of extracellular products that aid in survival and increased virulence [44]. We hypothesized that distinct virulence traits of ocular *P. aeruginosa* strains would be associated with increased antibiotic resistance in patients with positive *P. aeruginosa* cultures, and as expected we found that 35 out of the 46 *P. aeruginosa* strains had more than two virulence factors. Factor analysis of the measured bacterial variables revealed interrelationships between several of these phenotypes. In our study, the growth curve analysis showed that the *MDR-PA* strains had shorter doubling time, especially at later time points, along with increased swarming motility, and though this association was not statistically significant, it explains its longer persistence in the host. Similarly, pyoverdine which is said to play a critical role in the pathogenesis of host infection by *P. aeruginosa* by removing ferric iron from the host causing mitochondrial damage and compromising ATP production [45] was significantly associated with *MDR-PA* group. This is in agreement with findings by Rodulfo et al. [6] and Finlayson [7] wherein pigment production was reported to be significantly associated with MDR expression along with elastase, protease, siderophore and DNase activity. To date, considerable number of studies have shown the potential importance of pyocyanin in the virulence and pathogenicity of pseudomonal infections [46, 47]. In the present study, ocular *MDR-PA* strains showed a significant association with pyocyanin production.

Our experiments also show that protease production had a role in increased virulence of resistant strains and the XDR group showed higher activity compared to MDR strains. While catalase activity has been demonstrated to be essential for the intracellular survival of bacteria such as *Mycobacterium tuberculosis* [48], our study did not find any association of catalase and urease activity with antibiotic profile. We did however, find a correlation between resistance patterns and phospholipases activity which is reported to play an important role in host cell penetration, cell lysis and are active component of bacterial toxins. On the contrary, other studies have shown association of catalase with virulence. This enzyme has been demonstrated to be an essential factor for the intracellular survival of few bacteria such as *Mycobacterium tuberculosis* [49], *Campylobacter jejuni* [50] and *Helicobacter pylori* [51]. Similarly, Urease activity has been shown to be an important pathogenic factor for the bacteria *Helicobacter pylori* and *Proteus mirabilis* [52–54]. It is also known to be involved in a series of processes that allow bacteria to colonize and induce a strong inflammatory response in the gastric epithelium [55]. Citrate is known important activator of master regulators expression of virulence factors, central metabolism, iron acquisition, and bacterial virulence of *S. aureus* [56].

Biofilm is another reported cause of multi-drug resistance in *P. aeruginosa* [57] and our results were in agreement with previous studies wherein production of biofilm was significantly associated with MDR and XDR strains. An earlier study on *E.coli* strains had suggested that although the virulence of an organism cannot be predicted accurately on the basis of its measurable phenotypes, the presence of multiple virulence factors increases the virulence of the organisms [58], and along with host conditions decrease the need for multiple virulence factors in the strains leading to serious infections [59]. Our study is in agreement with Subedi et al. [45] who reported that virulence factors, extracellular products including proteases, and the ability to produce biofilm may explain the poor visual prognosis in *P. aeruginosa* endophthalmitis despite rapid antibiotic therapy [60]. The most important extracellular factors of *P. aeruginosa* include exo-S, exo-U, exo-A. Each of the aforementioned factors are known to have toxic effect on mammalian cells [61, 62]. In the present study, we found that exo-A is associated with multidrug resistant not exo-S and exo-U. An earlier study by wolf et al. [63] has shown that the exoA-deficient mutants exhibit virulence 20 times less than the wild type strain in the mouse models.

Principal component analysis and heat map data reiterates the unique virulence profile of *MDR-PA* strains compared to *S-PA* group and these five factors (pyoverdine production, biofilm ability, protease and phospholipase activity) could thus be used as independent predictors of resistant profiles and virulence. Several limitations however, exist in our study. Firstly, these experiments were measured in vitro of *P. aeruginosa* isolated from primary clinical specimens, although these phenotypes were reproducible upon repeat assay, we only determined potential of expression of virulence factors under defined in vitro conditions, and not actually expressed in the host. Secondly, the visual outcome in resistant strains was not correlated with antibiotic resistance in our clinical practice. Nonetheless, our findings would be more representative of patients with positive cultures for *P. aeruginosa* seen in tertiary eye care hospitals and may aid in clinician decision-making in such a setting.

## Conclusion

While antibiotic resistance is multifactorial, recognition of virulent strains by phenotypic characterization, is easier and allows immediate institution of appropriate therapy. To the best of our knowledge, this study was the first investigation regarding the phenotypic virulent characteristics amongst susceptible and resistant strains of *P. aeruginosa* in India. Further studies would focus to understand the genotypic characteristics involved in MDR-*P. aeruginosa* strains, which would aid in developing a rapid signature biomarker for resistant strains.

## Data Availability

The datasets analyzed during the current study are available from the corresponding author on reasonable request.
